# Extraction and purification of total flavonoids from *Eupatorium lindleyanum* DC. and evaluation of their antioxidant and enzyme inhibitory activities

**DOI:** 10.1002/fsn3.1999

**Published:** 2021-04-03

**Authors:** Chao Li, Shanglong Chen, Jin Sha, Jue Cui, Juping He, Junning Fu, Yingbin Shen

**Affiliations:** ^1^ College of Food and Bioengineering Xuzhou University of Technology Xuzhou China; ^2^ Department of Food Science and Engineering Jinan University Guangzhou China; ^3^ School of Life Sciences Guangzhou University Guangzhou China

**Keywords:** antioxidant activities, enzyme inhibitory activities, *Eupatorium lindleyanum* DC., total flavonoids, ultrasonic–microwave synergistic extraction

## Abstract

The health benefits and promising medical treatment potential of total flavonoids from *Eupatorium lindleyanum* DC. (TFELDC) have been recognized. The process parameters of extracting total flavonoids from *Eupatorium lindleyanum* DC. by ultrasonic–microwave synergistic extraction (UMSE) were optimized, and they were purified by AB‐8 macroporous resin in the current study. In addition, the antioxidant and enzyme inhibitory activities of the purified TFELDC (PTFELDC) were evaluated. The results showed that the optimal parameters of UMSE were as follows: ethanol volume fraction 71.5%, *L*/*S* ratio 12.2 ml/g, microwave power 318 W, and extraction time 143 s. After TFELDC were purified by AB‐8 macroporous resin, the total flavonoid contents of PTFELDC increased from 208.18 ± 1.60 to 511.19 ± 3.21 mg RE/g FDS. Compared with TFELDC, the content of total flavonoids in PTFELDC was increased by 2.46 times. The antioxidant activities of PTFELDC were assessed using DPPH radical, superoxide anion radical, reducing power, and ferric reducing antioxidant power assays, and the IC_50_ values were found to be 37.13, 19.62, 81.22, and 24.72 μg/ml, respectively. The enzyme inhibitory activities of PTFELDC were measured using lipase, α‐amylase, α‐glucosidase, and acetylcholinesterase assays with the IC_50_ values 1.38, 2.08, 1.63, and 0.58 mg/ml, respectively. By comparing with their positive controls, it was found that PTFELDC had good antioxidant activities, and lipase, α‐amylase, and α‐glucosidase inhibitory activities, However, the acetylcholinesterase inhibitory activity was relatively weaker. These results suggested that PTFELDC have a promising potential as natural antioxidant, antilipidemic, and hypoglycemic drugs used in functional foods or pharmaceuticals.

## Graphical abstract

1. The parameters for ultrasonic–microwave synergistic extraction (UMSE) of total flavonoids from *Eupatorium lindleyanum* DC. **(**TFELDC**)** were optimize using response surface model. 2. TFELDC were purified by AB‐8 macroporous resin. 3. Antioxidant and enzyme inhibitory activities of the purified TFELDC **(P**TFELDC**)** were evaluated.

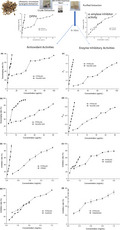

## 1 INTRODUCTION

*Eupatorium lindleyanum* DC., a traditional Chinese herb, belongs to compositae family, contains such pharmacological active ingredients such as flavonoids, volatile oil, and sesquiterpenes, and has been mainly applied for the treatment of cough, tracheitis, tonsillitis, and hypertension, etc.(Ji et al., 2008; Ye et al., 2008). Extracts of *Eupatorium lindleyanum* DC. have shown antimicrobial, antihistamine, and anti‐inflammatory activities, and protective effects acute lung injury (Chu, Ren et al., 2016; Chu, Yao et al., 2016; Ji et al., 2008).

In recent studies, the multitude of biological activities (antioxidant, enzyme inhibitory, antitumor, and anti‐inflammatory effects) of total flavonoid in plants has been discovered and proved (Alara et al., 2018; Liu et al., 2020; Martinez‐Gonzalez et al., 2019; Xavier‐ravi et al., 2019; Zhang, Wang et al., 2017).

And due to the advantages of low toxic and side effects compared with the chemically synthesized drugs, both the crude and purified total flavonoids in plants have attracted attention in nutraceutical and pharmaceutical resource researches (Zhang, Lie et al., 2017). However, compared with the other plants, the extraction and purification of total flavonoids from *Eupatorium lindleyanum* DC. (TFELDC) and the evaluation of their antioxidant and enzyme inhibitory activities have been rarely study, which may lead to the negative impact on their development and utilization.

Ultrasonic and microwave radiations have been well known by their effect in accelerating the extracting process and improving the bioactivity of the extraction (Ameer et al., 2017; Ben Ticha et al., 2017; Hsieh et al., 2016; Sökmen et al., 2017). The interest on applying sonochemistry to natural product extraction has increased because of its advantages (e.g., reduction in extraction time, saving in energy, and increased yield) (Rodrigues et al., 2008). Microwave can heat the extracts quickly and accelerate the extraction process for adsorption and desorption of the targeted compounds from matrix, while its disadvantage is inhomogeneous heating effect (You et al., 2014). Hence, combining microwave with ultrasonic extraction is a complementary technique and may present some more advantages. At present, UMSE has been applied to the extraction of polysaccharides, volatile oil, alkali, and flavonoids and compared with other extraction methods, such as soak extraction, heat extraction, ultrasonic‐assisted extraction, and microwave‐assisted extraction, which has the following merits: high extraction rate, fast speed, simple technics, pollution‐free, etc (Cheng et al., 2011; Jiang et al., 2019; Sun et al., 2019; Wang et al., 2018).

In this study, UMSE was applied as a rapid method of extraction of TFELDC, and AB‐8 macroporous resin was employed in the purification of TFELDC to achieve the purified TFELDC (PTFELDC). In addition, the antioxidant and enzyme inhibitory activities of PTFELDC were evaluated using DPPH radical, superoxide anion radical, reducing power, and ferric reducing antioxidant power assays, and lipase, α‐amylase, α‐glucosidase, and acetylcholinesterase assays. Findings in this study are helpful to obtain an effective and nontoxic natural antilipidemic, hypoglycemic, and antitumor source for functional foods and pharmaceuticals.

## 2 MATERIALS AND METHODS

### 2.1 Plant material

*Eupatorium lindleyanum* DC. was purchased from Bozhou Haiyitang medicines procurement Co., Ltd. and identified by Prof. Weidong Wang, Department of Food Sciences, Xuzhou University of Technology on 20 March 2018, which was taxonomically authenticated and deposited with voucher number no. 20180322–2 at the herbarium of Food Department, Xuzhou University of Technology, Xuzhou. It was dried in an oven (GZX‐9070MBE, Boxun, Shanghai, China) with air circulation at 60°C, and the dried material was ground into the fine and homogeneous powder using a grinder (WKX‐160, Jingcheng, Qingzhou, Shandong, China). Then, the sample powder was sieved through a 60 mesh sieve and kept in a sealed plastic bag at room temperature prior to use.

### 2.2 Chemicals

Lipase from porcine pancreatic (Type II, EC 3.1.1.3; L3126), α‐amylase from porcine pancreas (Type VI‐B, EC 3.2.1.1, A3176), α‐glucosidase from Saccharomyces cerevisiae (EC 3.2.1.20, G3651), and acetylcholinesterase from Electrophorus electricus (Type VI‐S, EC 3.1.1.7, C3389) were purchased from Sigma‐Aldrich Co., Ltd. Rutin (≥98%), ascorbic acid (≥98%), *p*‐nitrophenyl palmitate (*p*‐NPP), 3,5‐dinitrosalicylic acid (DNS), *p*‐nitrophenyl‐α‐D‐glucopyranoside (α‐p‐NPG), 5,5'‐dithiobis(2‐nitrobenzoic acid) (DTNB), acetylthiocholine iodide, and orlistat were purchased from Hefei Biobomei Biotechnoligy Co., Ltd.. Acarbose was purchased from Nanjing Dulai Biotechnology Co., Ltd.. *p*‐(Dimethylamino)cinnamaldehyde (*p‐*DMACA) and galantamine were purchased from Shanghai Yuanye Biotechnology Co., Ltd.. All other chemicals used were of analytical grade and procured from Sinopharm Chemical Reagent Co., Ltd. AB‐8 macroporous resin was purchased from Anhui Sanxing Resin Technology Co., Ltd.

### 2.3 Ultrasonic–microwave synergistic extraction (UMSE)

The ultrasonic–microwave synergistic extraction apparatus (CW‐2000, Xintuo) equipped with an ultrasonic control device (power from 10 to 800 W) and a microwave control device (power 50 W, frequency 40 kHz). 4.0 g of the sample powder was weighed exactly and placed in a 100 ml quartz extraction cell equipped with reflux system. After the apparatus was turned on, extraction time was counted and the extraction was carried out continuously at the preset parameters. When the extraction was completed, and the extracts were collected, filtrated, and fixed for the further determination of total flavonoid content. After the analysis, the remaining extract was concentrated, freeze‐dried, and stored at −20°C.

### 2.4 Experimental design

The effects of ethanol volume fraction, liquid‐to‐solid ratio (*L*/*S* ratio), microwave power, and extraction time on the extraction yield of TFELDC were firstly investigated. And then, on the basis of results above, the Box–Behnken design (BBD) (Samaram et al., 2015) with four independent variables at three levels was used to optimize the extraction conditions of UMSE of TFELDC. The levels of the three factors were as follows: ethanol volume fraction (*x*_1_) of 70%, 80%, and 90%; *L*/*S* ratio (*x*_2_) of 10, 12, and 14 ml/g; microwave power (*x*_3_) of 200, 300, and 400 W; and extraction time (*x*_3_) of 120, 140, and 160 s. The extraction yield was taken as the dependent variable.

The generalized second‐order polynomial model used in the response surface analysis was as follows:

(1) $$\text{Extraction yield}\;\left(\%\right)=\beta_{0}+\sum_{i=1}^{4}\beta_{i}x_{i}+\sum_{i=1}^{4}\beta_{\mathrm{ii}}x_{i}^{2}+\sum_{i=1}^{3}\sum_{j=i+1}^{4}\beta_{\mathrm{ij}}x_{i}x_{j}$$

where *β*_0_ is the constant coefficient; *β*_i_, *β*_ij_, and *β*_ii_ are the regression coefficients for the linear, interaction, and quadratic terms; *x*_i_ and *x*_j_ are the independent variable actual value.

### 2.5 Determination of extraction yield

The extraction yield of TFELDC was calculated according to the following equation by the method of Alara et al. (2018) with some modifications.

(2) $$\text{Extraction yield (\textbackslash{}\% ) =}\;\frac{W_{e}}{W_{t}}\times 100$$

where *W*_e_ is the mass of rutin equivalents extracted in the solution, and *W*_t_ is the mass of *E. lindleyanum* DC.

### 2.6 Purification of TFELDC

The purification of TFELDC was carried out according to the method of Chen et al. (2018) with some modifications. Dynamic adsorption and desorption tests were carried out on a column (2.6 × 60 cm) wet‐packed with AB‐8 macroporous resin, and the bed volume (BV) was 290 ml. The dynamic adsorption tests were performed as follows: The pH value of sample solution (2 g of crude extract dissolved in 1,000 ml of deionized water) was adjusted to 4.6 with 1 M hydrochloric acid and loaded onto the column at a flow rate of 1 BV/h. The dynamic desorption tests were performed as follows: After sample being loaded, distilled water was used for elution until the eluent was colorless, and then the column was eluted by 3.5 BV of 70% ethanol at a flow rate of 0.5 BV/h. At last, the elution solution was collected, concentrated, and lyophilized for studying its phytochemical properties and biological activities in vitro, respectively.

### 2.7 Determination of total flavonoid content

The total flavonoid content was determined according to the colorimetric method developed by Benabderrahim et al. (2019) with minor modifications. Briefly, 5.4 ml of different concentrations of rutin and 0.3 ml of 5% (w/v) sodium nitrite were mixed in a 10‐ml colorimetric tubes. After the mixture was incubated for 6 min in the dark at 37°C. Following the addition of 0.3 ml of 10% (w/v) aluminum nitrate, the mixture was incubated for 6 min in the dark at 37°C. Subsequently, 4 ml of 1 M sodium hydroxide was added into them, and the mixture was incubated for 10 min in the dark at 37°C. The absorbance of the mixture at 510 nm was measured using a UV‐Vis spectrophotometer (Shanghai Spectrum Instrument Co. Ltd.). The total flavonoid contents in TFELDC or PTFELDC were expressed as mg rutin equivalents per gram of freeze‐dried sample (mg RE/g FDS) through the calibration curve of rutin.

### 2.8 Antioxidant assays

#### 2.8.1 DPPH radical scavenging assay

The DPPH radical scavenging activity of PTFELDC was assessed using the method in the research by Chen and Huang (2019) with minor modifications. Briefly, 50 µl of different concentrations of PTFELDC was mixed with 150 µl of 0.15 mM DPPH (in ethanol) in 96‐well plates. After the mixture was incubated for 60 min in the dark at 37°C, the absorbance of the mixture at 517 nm was measured using a microplate reader (Synergy H1, Bio‐Tek). Ascorbic acid was used as a positive control. The scavenging rate was calculated as follows:

(3) $$\text{Scavenging rate (\textbackslash{}\% ) = (1 -}\;\frac{A_{i}‐A_{j}}{A_{c}})\times 100$$

where *A*_i_ is the absorbance in the presence of the sample, *A*_j_ is the absorbance without DPPH, and *A*_c_ is the absorbance of the control (without sample).

#### 2.8.2 Superoxide anion radical scavenging assay

The superoxide anion radical scavenging activity of the samples was assessed using the method in the research by Sfahlan et al. (2009), Zhang et al. (2018) and Zhang et al. (2015) with minor modifications. Briefly, 20 µl of sample solutions and 100 µl of Tris‐HCl buffer (50 mM, pH 8.20) were added in 96‐well plates to achieve the final concentrations of 5, 10, 15, 20, 25, 30, 35, 40, 45, and 50 µg/ml, respectively. Subsequently, the mixture was incubated in the dark at 37°C for 20 min. Next, 8 µl of pyrogallol (3 mM of pyrogallol in 10 mM of HCl), which was also preincubated at 37°C for 5 min, was injected to the 96‐well plates. And then, the mixture was incubated in the dark at 37°C for 5 min. After incubation, 32 µl of HCl (1 M) was added into the mixture promptly to terminate the reaction. Finally, the absorbance of the mixture was measured at 320 nm using a microplate reader. Ascorbic acid was used as a positive control. The scavenging rate was calculated as Equation 3.

#### 2.8.3 Reducing power (RP) assay

The reducing power of PTFELDC was assessed using the method in the research by Locatelli et al. (2010) with minor modifications. Briefly, 10 µl of different concentrations of PTFELDC and 25 µl of 0.2 M phosphate buffer (PBS, pH 6.6) was mixed with 25 µl of 1% (w/v) potassium ferricyanide in 96‐well plates. After the mixture was incubated for 30 min at 37°C, 25 µl of 10% (w/v) trichloroacetic acid was added to terminate the reaction. Subsequently, 85 µl of distilled water and 17 µl of 0.1% (w/v) ferric chloride were added to the above mixture, and the absorbance of the mixture at 700 nm was measured using a microplate reader. Ascorbic acid was used as a positive control. The reducing power effect was calculated as follows:

(4) $$A_{\text{rp}}=A_{i}‐A_{c}$$

where A_rp_ is the reducing power, *A*_i_ is the absorbance in the presence of the sample, and *A*_c_ is the absorbance of the control (without sample).

#### 2.8.4 Ferric reducing antioxidant power (FRAP) assay

The antioxidant potential of PTFELDC was assessed by using the FRAP assay based on the research of Zdunić et al. (2020) with minor modifications. Briefly, the FRAP reagent was prepared by mixing 10 mM TPTZ (in 40 mM HCl), 20 mM ferric chloride, and acetate buffer (0.3 M, pH 3.6) at 1:1:10 (v/v/v). Then, 185 µl of freshly prepared FRAP reagent was mixed with 15 µl of different concentrations of PTFELDC in 96‐well plates. After the mixture was incubated for 10 min in the dark at 37°C, the absorbance at 593 nm was measured using a microplate reader. Ascorbic acid was used as a positive control. *A*_frap_ was the ferric reducing antioxidant power, which was calculated as Equation (4).

### 2.9 Enzyme inhibitory assays

#### 2.9.1 Lipase inhibitory assay

The lipase inhibitory activity of PTFELDC was measured using the method in the research by Spínola et al. (2018) with minor modifications. Briefly, 50 μl of different concentrations of PTFELDC was mixed with 50 μl of 1.2 U/ml pancreatic lipase solution in PBS (0.1 M, pH 8.0). After the mixture was incubated for 10 min in the dark at 37°C, 100 μl of 0.2 mM *p‐*NPP (in PBS), which was preincubated at 37°C for 10 min, was added. Subsequently, the mixture was incubated for 20 min in the dark at 37°C. The absorbance at 405 nm was measured using a microplate reader. Orlistat was used as a positive control. The inhibitory rate was calculated as follows:

(5) $$\text{Inhibition rate (\textbackslash{}\% ) = (1 -}\;\frac{A_{i}‐A_{j}}{A_{c}‐A_{d}})\times 100$$

where *A*_i_ is the absorbance in the presence of sample, enzyme, and substrate, *A*_j_ is the absorbance in the presence of sample, PBS (instead of enzyme), and substrate; *A*_c_ is the absorbance in the presence of PBS (instead of sample), enzyme, and substrate; *A*_d_ is the absorbance in the presence of PBS (instead of sample and enzyme) and substrate.

#### 2.9.2 α‐amylase inhibitory assay

The α‐amylase inhibitory activity of PTFELDC was measured using the method in the research by Guo et al. (2019) with minor modifications. Briefly, 700 μL of varying concentrations of sample solution was mixed with 600 μL of 1% (m/v) soluble starch in test tubes. After the mixture was incubated at 37°C in the dark for 5 min, 200 μL of 20 U/ml α‐amylase solution (in deionized water), which was preincubated at 37°C in the dark for 5 min, was added and incubated at 37°C in the dark for 5 min. Subsequently, 500 μL of 1% (m/v) dinitrosalicylic acid reagent was added and the mixture was incubated at 100°C in the dark for 5 min. The mixture was cooled down to room temperature and diluted to 10 ml with deionized water. Finally, 200 μL from each sample was seeded in a 96‐well plate, and the absorbance at 540 nm was measured using a microplate reader. Acarbose was used as a positive control. The inhibitory rate was calculated as Equation (5).

#### 2.9.3 α‐glucosidase inhibitory assay

The α‐glucosidase inhibitory activity of PTFELDC was measured using the method in the research by Gutiérrez‐Grijalva et al. (2019) with minor modifications. Briefly, 40 μl of different concentrations of PTFELDC and 40 μl of 5 mM *p*‐nitrophenyl α‐D‐glucopyranoside (*p*‐NPG) in PBS (0.1 M, pH 7.0) were mixed and incubated for 10 min in the dark at 37°C. Then, 20 μl of 40 U/ml a‐glucosidase (in PBS), which was preincubated at 37°C for 5 min, was added to the above mixture. Subsequently, the mixture was incubated for 20 min in the dark at 37°C. Finally, the reaction was terminated by the addition of 100 μl of 0.3 mM sodium carbonate, and the absorbance at 405 nm was measured using a microplate reader. Acarbose was used as a positive control. The inhibitory rate was calculated as Equation (5).

#### 2.9.4 Acetylcholinesterase inhibitory assay

The acetylcholinesterase inhibitory activity of PTFELDC was measured using the method in the research by Li et al. (2019) with minor modifications. Briefly, 100 μl of different concentrations of PTFELDC was mixed with 20 μl of 0.2 U/ml acetylcholinesterase in PBS (0.1 M, pH 8.0). After the mixture was incubated for 15 min in the dark at 37°C, 40 μl of 1 mM DTNB (in PBS) and 40 μl of 1.875 mM acetylthiocholine iodide (in PBS), which were preincubated at 37°C for 5 min, were added. Subsequently, the mixture was incubated for 20 min in the dark at 37°C. The absorbance at 405 nm was measured using a microplate reader. Galantamine was used as a positive control. The inhibitory rate was calculated as Equation (5).

#### 2.9.5 Statistical analysis

All experiments were performed in triplicate and expressed as means ± standard deviation (*SD*). The experimental data from single‐factor experiments were analyzed by one‐way analysis of variance (ANOVA) and Duncan's multiple comparisons performed by SPSS V18.0 software (IBM Co.), and the figures were plotted using Origin V8.0 software (Origin Lab CO.). The statistical analysis of the experimental results of Box–Behnken design was carried out, and 2‐D contour plots and 3‐D response surface plots were plotted using Design‐Expert V8.0.6 software (State‐Ease, Inc.). IC_50_ values were calculated by SPSS V18.0 software.

## 3 RESULTS AND DISCUSSION

### 3.1 Single‐factor experiment

#### 3.1.1 Effects of ethanol volume fraction

**FIGURE 1:**
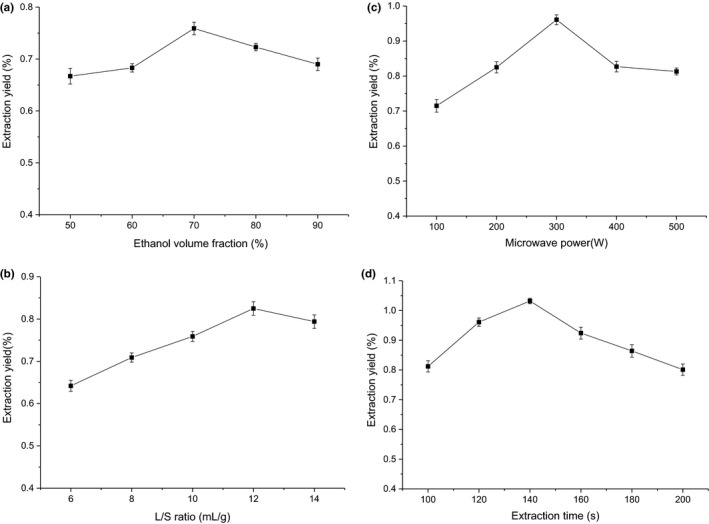
Effects of different extraction parameters on extraction yield of total flavonoids. (a) Effects of ethanol volume fraction. (b) Effects of *L*/*S* ratio. (c) Effects of microwave power. (d) Effects of extraction time

Different concentrations of solvent have an effect on the polarity of the solvent, so it is very important to find a optimal concentration in order to get a higher extraction rate (Yuan et al., 2014). In general, the solubility of total flavonoids in a certain concentration of ethanol is excellent. Therefore, ethanol was applied to extract TFELDC and the results were depicted in Figure 1a. The extraction rates of TFELDC firstly increased from 0.667 ± 0.015% to 0.759 ± 0.012% and then declined to 0.690 ± 0.012% with the increase in ethanol volume fraction from 60% to 100%. This was because that the polarity of the mixer changes when water and ethanol were mixed together. At a level of 80% (v/v) ethanol, the polarities of the mixer and total flavonoids were in close proximity. Based on the theory of "similarity and intermiscibility," the extraction rate reached its peak value under the conditions. Based on the current results, 80% was considered to be the optimum ethanol volume fraction in the present experiment.

#### 3.1.2 Effects of *L*/*S* ratio

*L*/*S* ratio is an important extraction parameter affecting the extraction rate of flavonoids (Wang et al., 2010), and the results of the effects of *L*/*S* ratio on the extraction rates of TFELDC were depicted in Figure 1b. The extraction rates of TFELDC increased from 0.642 ± 0.013% to 0.825 ± 0.016%, as the *L*/*S* ratios increased from 6:1 to 12:1 ml/g, but a further increase in the *L*/*S* ratio decreased the extraction rate to 0.794 ± 0.016%. This was because that a larger *L*/*S* ratio implied greater concentration difference between the plant material and the exterior extraction solvent, and so the diffusion of target compounds were more rapidly (Zhu et al., 2015). Additionally, the higher *L*/*S* ratio could reduce the viscosity of the extraction solvent, which can make the diffusion of target components easier and facilitate acoustic cavitation (Tsiaka et al., 2015). However, when *L*/*S* ratio was too high, both of the microwave and ultrasonic intensity imposed on the average vegetal tissue were weakened, which was not conducive for the fragmentation of raw material (Xu et al., 2014). At the same time, from an economic perspective, using a large amount of extraction solvent was not considered cost‐effective due to the high operating cost of extraction solvents and energy consumption (Yang et al., 2013). So, *L*/*S* ratio of 12:1 ml/g was selected for further study.

#### 3.1.3 Effects of microwave power

Microwave power significantly influenced the target components yield in the extraction process (Dahmoune et al., 2014). As shown in Figure 1c, with the increase in microwave power, the extraction yields of TFELDC increased. This was because microwave can crack the cell wall, disrupt the tissue structure of material, and accelerate the dissolution of target components (Ji et al., 2007), resulting in high extraction yield of TFELDC in earlier stage of UMSE in the study. However, when microwave power was above 300 W, the extraction yield declined. This was because overexposure under microwave irradiation resulted in local high temperature, which might decompose target components (Chen et al., 2017). From the above results, 300 W was chosen as the suitable microwave power in the further discussion.

#### 3.1.4 Effects of extraction time

The extraction time has also a significant effect on the extraction yield (Setyaningsih et al., 2015). In order to investigate the extraction efficiency and optimize the extraction process of UVSE, the extraction time was investigated and the results were depicted in Figure 1d. The extraction rates of TFELDC increased rapidly from 0.812 ± 0.019% to 1.032 ± 0.009%, when extraction time varied from 100 to 140 s, and reached a maximum at 140 s. However, the extraction rate was slowly decreased to 0.801 ± 0.019% with the prolonging of extraction time. This was because that as extraction time increases, both of microwave and ultrasonic wave could effectively disrupt the cell walls, leading to the release of target compounds into the exterior extraction solvent and the increase in extraction rates at the early period of extraction (Maran et al., 2015). But the exposure of microwave or ultrasonic treatment for long time caused the decomposition of certain sensitive bioactive compounds, which reduced the extraction rates (Chen et al., 2017; Xu et al., 2016). Consequently, it can be obtained that 140 s was chosen as the suitable extraction time.

### 3.2 Establishment of a model and analysis of response surface

#### 3.2.1 Fitting the model

A regression analysis was carried out to fit mathematical models to the experimental data (Table 1) aiming at an optimal region for the responses studied. The predicted model could be described by the following equation in terms of coded values:

(6) $$\text{Extraction yield}\;\left(\%\right)\;=-9.415+9.899\times 10^{-2}x_{1}+0.51x_{2}+3.428\times 10^{-3}x_{3}+4.543\times 10^{-2}x_{4}-2.25\times 10^{-4}x_{1}x_{2}+2.925\times 10^{-5}x_{1}x_{3}+2.5\times 10^{-6}x_{1}x_{4}+7.75\times 10^{-5}x_{2}x_{3}+4.562\times 10^{-4}x_{2}x_{4}+3.375\times 10^{-6}x_{3}x_{4}-7.407\times 10^{-4}x_{1}^{2}-2.358\times 10^{-2}x_{2}^{2}-1.089\times 10^{-5}x_{3}^{2}-1.817\times 10^{-4}x_{4}^{2}$$

**TABLE 1:** Experiment results of Box–Behnken

No.	*x*_1_	*x*_2_	*x*_3_	*x*_4_	Extraction yield (%, Actual Value)	Extraction yield (%, Predicted Value)
1	60	10	300	140	0.847	0.825
2	80	10	300	140	0.858	0.869
3	60	14	300	140	0.883	0.877
4	80	14	300	140	0.876	0.903
5	70	12	200	120	0.795	0.804
6	70	12	400	120	0.847	0.858
7	70	12	200	160	0.845	0.838
8	70	12	400	160	0.924	0.920
9	60	12	300	120	0.844	0.849
10	80	12	300	120	0.859	0.883
11	60	12	300	160	0.917	0.896
12	80	12	300	160	0.934	0.932
13	70	10	200	140	0.781	0.793
14	70	14	200	140	0.788	0.805
15	70	10	400	140	0.845	0.831
16	70	14	400	140	0.914	0.905
17	60	12	200	140	0.822	0.832
18	80	12	200	140	0.849	0.808
19	60	12	400	140	0.808	0.841
20	80	12	400	140	0.952	0.934
21	70	10	300	120	0.856	0.842
22	70	14	300	120	0.884	0.849
23	70	10	300	160	0.827	0.854
24	70	14	300	160	0.928	0.934
25	70	12	300	140	1.012	1.037
26	70	12	300	140	1.051	1.037
27	70	12	300	140	1.022	1.037
28	70	12	300	140	1.034	1.037
29	70	12	300	140	1.065	1.037

The significance of each coefficient was determined using the *F*‐value and *p*‐value in Table 2. The corresponding variables would be more significant if the absolute *F*‐value becomes greater and the *P*‐value becomes smaller. It was observed that the linear term of microwave power (*x*_3_) and all of the quadratics terms were significant at 0.001 level, the linear term of extraction time (*x*_4_) significant at 0.01 level, the linear term of ethanol volume fraction (*x*_1_) and *L*/*S* ratio (*x*_2_) significant at 0.05 level, and all of the interaction terms insignificant at 0.05 level. As shown in Table 2, the model was of great significance (*p* < .0001), the lack of fit (*p* = .2791 > 0.05) insignificant, the coefficient of determination (*R* ^2^) of the predicted model 0.9413 and the Adeq.Precision 12.296 (much larger than 4), which showed that the model was reliable.

**TABLE 2:** ANOVA results of quadratic regression model for response surface

Source	SS	*df*	MS	*F*‐value	*P*‐value	
Model	0.17	14	0.012	16.03	<0.0001	***
*x*_1_	3.571 × 10^–3^	1	3.571 × 10^–3^	4.71	0.0477	*
*x*_2_	5.590 × 10^–3^	1	5.590 × 10^–3^	7.37	0.0168	*
*x*_3_	0.014	1	0.014	18.47	0.0007	***
*x*_4_	7.008 × 10^–3^	1	7.008 × 10^–3^	9.24	0.0088	**
*x*_1_ *x*_2_	8.100 × 10^–5^	1	8.100 × 10^–5^	0.11	0.7486	
*x*_1_ *x*_3_	3.422 × 10^–3^	1	3.422 × 10^–3^	4.51	0.0519	
*x*_1_ *x*_4_	1.000 × 10^–6^	1	1.000 × 10^–6^	1.319 × 10^–3^	0.9715	
*x*_2_ *x*_3_	9.610 × 10^–4^	1	9.610 × 10^–4^	1.27	0.2792	
*x*_2_ *x*_4_	1.332 × 10^–3^	1	1.332 × 10^–3^	1.76	0.2062	
*x*_3_ *x*_4_	1.823 × 10^–4^	1	1.823 × 10^–4^	0.24	0.6315	
*x*_1_^2^	0.036	1	0.036	46.93	<0.0001	***
*x*_2_^2^	0.058	1	0.058	76.10	<0.0001	***
*x*_3_^2^	0.077	1	0.077	101.52	<0.0001	***
*x*_4_^2^	0.034	1	0.034	45.20	<0.0001	***
Residual	0.011	14	7.583 × 10^–4^			
Lack of Fit	8.777 × 10^–3^	10	8.777 × 10^–4^	1.91	0.2791	
Pure Error	1.839 × 10^–3^	4	4.597 × 10^–4^			
Cor Total	0.18	28				
		*R* ^2^ = 0.9413		Adeq.Precision = 12.296		

* Significant at 0.05 level

** Significant at 0.01 level

*** Significant at 0.001 level.

#### 3.2.2 Analysis of response surface

**FIGURE 2:**
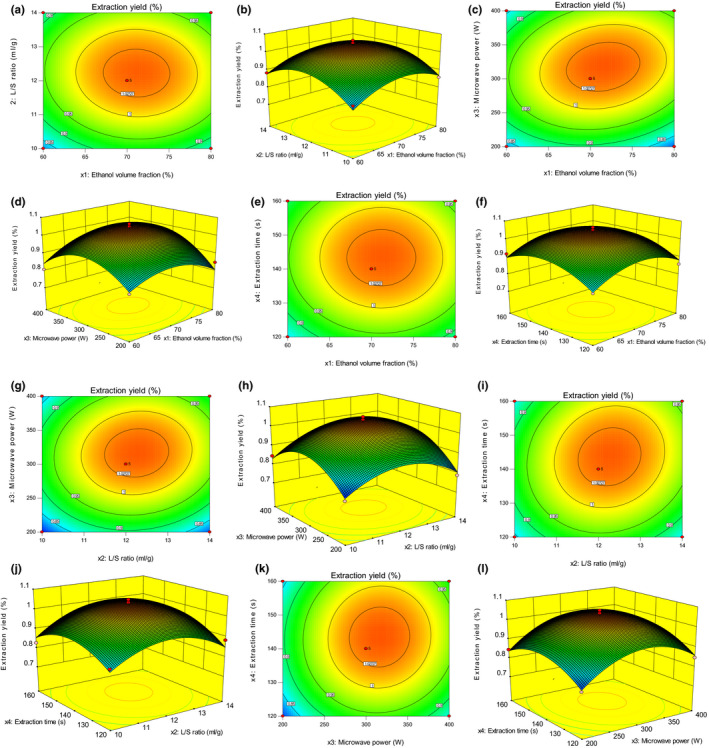
Contour and response surface of effects of two factors on extraction yield of total flavonoids. (a) Contour of effects of ethanol volume fraction and *L*/*S* ratio. (b) Response surface of effects of ethanol volume fraction and *L*/*S* ratio. (c) Contour of effects of ethanol volume fraction and microwave power. (d) Response surface of effects of ethanol volume fraction and microwave power. (e) Contour of effects of ethanol volume fraction and extraction time. (f) Response surface of effects of ethanol volume fraction and extraction time. (g) Contour of effects of *L*/*S* ratio and microwave power. (h) Response surface of effects of *L*/*S* ratio and microwave power. (i) Contour of effects of *L*/*S* ratio and extraction time. (j) Response surface of effects of *L*/*S* ratio and extraction time. (k) Contour of effects of microwave power and extraction time. (l) Response surface of effects of microwave power and extraction time

The regression model was able to predict the effects of the four factors on the extraction yield of TFELDC. The relationship between independent and dependent variables was illustrated in 2‐D contour plots and 3‐D response surface plots, which were generated by the model (Figure 2). Two variables were depicted in 3‐D response surface plots while the other two variables kept constant. The shape of 2‐D contour plots reflected the degree of the mutual effect. The oval suggested that the effect is significant while roundness insignificant. As shown in the Figure 2, the mutual effects between any two variables were insignificant at 0.05 level.

#### 3.2.3 Verification experiments

The optimal extraction conditions for achieving the maximal extraction yield of TFELDC obtained by the regression Equation (6) were as follows: ethanol volume fraction 71.5%, *L*/*S* ratio 12.2 ml/g, microwave power 318 W, and Extraction time 143 s. The predicted extraction yield was 1.044%. Under these conditions, the experimental extraction yield was 1.050 ± 0.006% (*n* = 3), which was well‐matched with the predicted value. The result indicated that the model was adequate for the extraction process.

### 3.3 Purification of TFELDC

AB‐8 macroporous resin, a type of weak polar resin, has been extensively applied in the purification of plant flavonoids, because of its physicochemical stability, adsorption/desorption selectivity, and recyclability (Gu et al., 2016; Wang & Wang, 2019). After TFELDC were purified by AB‐8 macroporous resin, the total flavonoid contents of PTFELDC increased from 208.18 ± 1.60 to 511.19 ± 3.21 mg RE/g FDS. The content of total flavonoids was increased by 2.46 times. Therefore, it could be concluded that the purification of TFELDC by AB‐8 macroporous resin was an effective method. The results were in agreement with earlier studies (Hamed et al., 2019; Zhang et al., 2011).

### 3.4 Antioxidant activities

#### 3.4.1 DPPH radical scavenging activity

**FIGURE 3:**
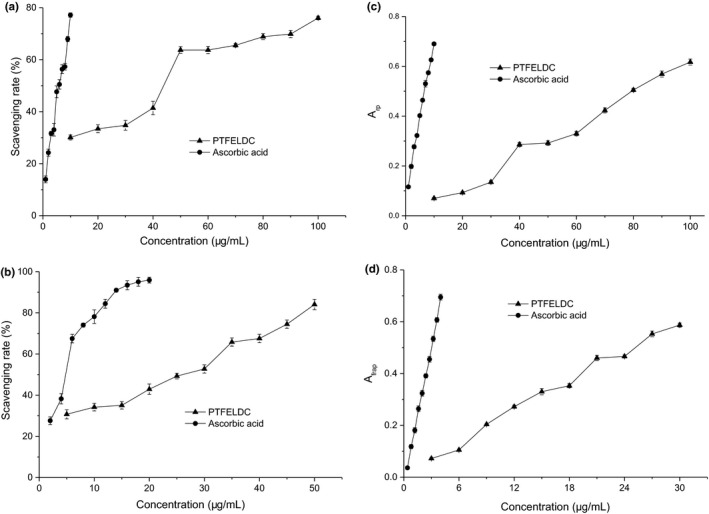
Antioxidant activities. (a) DPPH radical scavenging activity. (b) Superoxide anion radical scavenging activity. (c) Reducing power activity. (d) Ferric reducing antioxidant power activity

DPPH radical scavenging assay, based on the reduction of DPPH solution in the presence of a proton‐donating substance, has been extensively used to evaluate the free radical scavenging ability of varied samples (Chen et al., 2012). The scavenging activity of PTFELDC on DPPH radicals was shown in Figure 3a with ascorbic acid as a control standard. It was obvious that the scavenging rates of PTFELDC on DPPH radicals were positively correlated with increasing concentrations. At the concentration of 10 μg/ml, PTFELDC showed the scavenging rates of 30.12 ± 0.94% on DPPH radical, and at 100 μg/ml, the scavenging rate increased to 76.08 ± 0.70%. The parameter used to compare the DPPH radical scavenging activity of PTFELDC and ascorbic acid is IC_50_ value, defined as the concentrations of the sample at which the scavenging rate reaches 50% (Guo et al., 2011) and calculated by the probit regression (de Lima et al., 2015). Practically, a lower IC_50_ value corresponds to stronger antioxidant activity of tested sample (Kozarski et al., 2011). The IC_50_ value of PTFELDC was 37.13 μg/ml, which was 6.84 times that of ascorbic acid (5.43 μg/ml). Although the IC_50_ value for the standard ascorbic acid in the assay is significantly different from those (90 or 140 μg/ml) in the literature reported by Ng et al. (2020) and Ng et al. (2020), it is basically equivalent to those (5.28 or 4.11 μg/ml) in the literature reported by Dong et al. (2019) and Ng et al. (2020). This is because that the IC_50_ for the standard ascorbic acid is related to the determination conditions (such as temperature, time, and optical path), calculation method (such as whether to remove the background and the choice of experimental point).

#### 3.4.2 Superoxide anion radical scavenging activity

Superoxide radical is a powerful oxidizing agent that can react with biological membranes and induce tissue damage. It also decomposes to singlet oxygen, hydroxyl radical, or hydrogen peroxide (Siriwardhana & Shahidi, 2002). The scavenging activity of PTFELDC on superoxide anion radicals was shown in Figure 3b with ascorbic acid as a control standard. It was shown that the scavenging rates of PTFELDC on superoxide anion radicals were positively correlated with increasing concentrations. At the concentration of 5.0 μg/ml, PTFELDC showed the scavenging rate of 30.72 ± 2.19% on superoxide anion radical, and at 50.0 μg/ml, the scavenging rate increased to 84.06 ± 2.51%. The IC_50_ value of PTFELDC was 19.62 μg/ml, which was 4.63 times that of ascorbic acid (4.24 μg/ml).

#### 3.4.3 RP activity

The reducing power assay measures the electron‐donating ability of antioxidants by the potassium ferricyanide reduction method. The presence of antioxidants would result in the reduction capacity of potassium ferricyanide (Fe^3+^) to potassium ferrocyanide (Fe^2+^), which further reduces the production of ferric ferrocyanide. The reducing capacity of samples may serve as an index to evaluate the potential antioxidant properties (Yang et al., 2011). As shown in Figure 3c, the *A*_rp_ values of PTFELDC showed a concentration‐dependent manner with increasing concentrations. At the concentration of 10 μg/ml, PTFELDC showed the *A*_rp_ was 0.070 ± 0.004, and at 100 μg/ml, the *A*_rp_ increased to 0.617 ± 0.012. IC_50_ value is defined as the effective concentration at which the absorbance is 0.5 for reducing power and was calculated by interpolation from linear regression analysis (Guo et al., 2011). The IC_50_ values of PTFELDC and ascorbic acid were 81.22 and 6.78 μg/ml, which indicated that PTFELDC has better reducing power.

#### 3.4.4 FRAP activity

The FRAP activity measures the reduction capacity of the TPTZ‐Fe(III) complex to its ferrous TPTZ‐Fe(II) form using antioxidants (Sokamte et al., 2019). As shown in Figure 3d, the *A*_frap_ values of PTFELDC showed a concentration‐dependent manner with increasing concentrations. At the concentration of 3 μg/ml, PTFELDC showed the *A*_frap_ was 0.072 ± 0.003, and at 30 μg/ml, the *A*_frap_ increased to 0.587 ± 0.008. The IC_50_ values of PTFELDC and ascorbic acid were 24.72 and 2.63 μg/ml, which indicated that PTFELDC has better ferric reducing antioxidant power.

### 3.5 Enzyme inhibitory activities

#### 3.5.1 Lipase inhibitory activity

**FIGURE 4:**
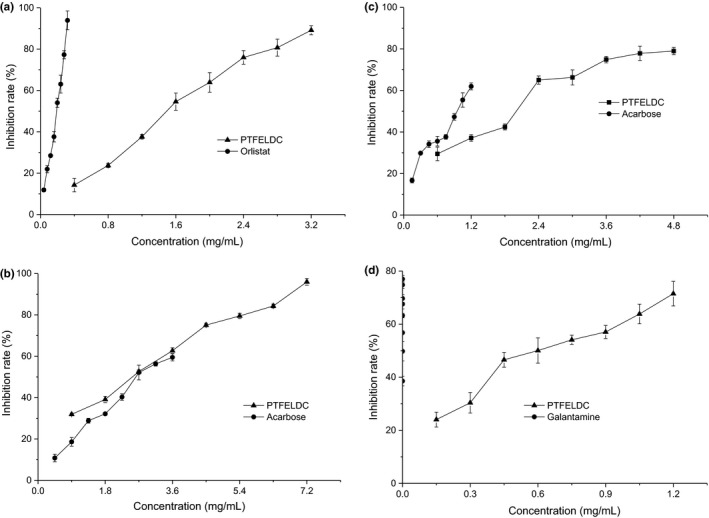
Enzyme inhibitory activities. (a) Lipase inhibitory activity. (b) α‐Amylase inhibitor activity. (c) α‐Glucosidase inhibitory activity. (d) Acetylcholinesterase inhibitory activity

Lipase is responsible for the metabolism of triglycerides, which is the key enzyme in the process of fat hydrolyzate in organism (Spínola et al., 2020). When the activity of lipase is too high, the organism will produce excessive monoglycerides and fatty acids, which will lead to the hyperlipidemia. By inhibiting the activity of lipase, the lipase inhibitor can reduce the content of lipid hydrolyzate and accelerate lipid exclusion from the body so as to achieve the goal of losing weight. At present, the commonly used lipase inhibitors are orlistat. However, the drug orlistat, a potent and specific long‐term gastrointestinal lipase inhibitor, has serious side effects, such as oily stools, diarrhea, abdominal pain, and fecal spots (Filippatos et al., 2008). Therefore, it is hoped to discover new alternatives by scholars at home and abroad, which could increase the therapeutic effect and reduce the side effects. To this day, many natural plant extracts have been reported to possess lipase inhibitory activity (Herrera et al., 2019; Inthongkaew et al., 2017; Sosnowska et al., 2018; Świerczewska et al., 2019). The lipase inhibitory activity of PTFELDC was plotted in Figure 4a. The PTFELDC were able to inhibit the lipase activity dose‐dependently. When the concentrations of PTFELDC were in the range of 0.40–3.20 mg/ml, the effects of them, expressed as inhibition rates, increased from 14.26 ± 3.27% to 89.16 ± 2.15%. Orlistat used as a positive control exhibited the lipase inhibition rates from 11.89 ± 0.79% to 93.91 ± 4.54%, when its concentrations changed from 0.04 to 0.32 mg/ml. The results showed that the IC_50_ values of PTFELDC and orlistat were 1.38 and 0.16 mg/ml, respectively. However, this does not prevent PTFELDC from becoming a good lipase inhibitor.

#### 3.5.2 α‐amylase inhibitory activity

Inhibition of α‐amylase, an enzyme that plays a key role in digestion of starch, is considered as a strategy in the treatment of type II diabetes (Berrout et al., 2018). The α‐amylase inhibitory activity of PTFELDC was plotted in Figure 4b. The PTFELDC (from 0.90 to 7.20 mg/ml) were able to inhibit the α‐amylase activity dose‐dependently, and the inhibition rates of them increased from 31.93 ± 0.54% to 95.97 ± 1.61%. Acarbose used as a positive control exhibited the α‐amylase inhibition rates from 10.80 ± 1.86% to 59.49 ± 1.70%, when its concentrations changed from 1.49 to 3.60 mg/ml. The results showed that the IC_50_ values of PTFELDC and acarbose were 2.08 and 2.76 mg/ml, respectively. Compared with acarbose, PTFELDC displayed a superior inhibitory activity on α‐amylase, about 0.85 times higher. The IC_50_ value for the standard acarbose in the assay is significantly different from that (voglibose, 19.33 mg/ml) in the literature reported by Ng & See (2019), and it is basically equivalent to that (acarbose, 1.89 mg/ml) in the literature reported by Oyedeji‐Amusa & Ashafa (2019). This is because that the IC_50_ value for the standard is related to types of standard and the detection system.

#### 3.5.3 α‐glucosidase inhibitory activity

The α‐glucosidase located on the brush border surface membrane of intestinal cells is responsible for degrading oligosaccharides into monosaccharides, thus leading to an increase in blood glucose level (Baessa et al., 2019; Kim et al., 2010). Therefore, α‐glucosidase has been recognized as a potential targets for the management of diabetes (Beidokhti et al., 2020). The α‐glucosidase activity of PTFELDC was plotted in Figure 4c. The PTFELDC (from 0.60 to 4.80 mg/ml) were able to inhibit the α‐glucosidase activity dose‐dependently, and the inhibition rates of them increased from 29.46 ± 3.23% to 78.99 ± 1.71%. Acarbose used as a positive control exhibited the α‐glucosidase inhibition rates from 16.67 ± 1.18% to 61.96 ± 1.67%, when its concentrations changed from 0.15 to 1.20 mg/ml. The results showed that the IC_50_ values of α‐glucosidase activity inhibition of PTFELDC and acarbose were 1.63 and 0.94 mg/ml, respectively. The disparity between the effects of PTFELDC and acarbose on α‐glucosidase inhibitory activity was less than 2 times, so PTFELDC was a good α‐glucosidase inhibitor. The IC_50_ value for the standard acarbose in the assay is significantly different from that (voglibose, 10.47 mg/ml) in the literature reported by Ng and Rosman (2019), and it is basically equivalent to that (acarbose, 1.02 mg/ml) in the literature reported by Oyedeji‐Amusa and Ashafa (2019). The reason for this phenomenon is similar to that in α‐amylase inhibition assay.

#### 3.5.4 Acetylcholinesterase inhibitory activity

Acetylcholinesterase is a kind of transmitter hydrolase, which mainly exists in the postsynaptic membrane of human neuromuscular tissue (Oliveira et al., 2011). Its major biological function is to hydrolyze acetylcholine at the cholinergic synapse rapidly, prevent acetylcholine accumulation, and maintain the normal physiological function of the nervous system (Masuoka et al., 2019). Thus, inhibition of acetylcholinesterase has been considered as a promising approach for the treatment of Alzheimer's disease and possible therapeutic applications for the treatment of Parkinson's disease, aging, and myasthenia gravis (Bianco et al., 2015; Masondo et al., 2019). The acetylcholinesterase activity of PTFELDC was plotted in Figure 4d. The PTFELDC (from 150 to 1,200 μg/ml) were able to inhibit the acetylcholinesterase activity dose‐dependently, and the inhibition rates of them increased from 24.04 ± 2.79% to 71.48 ± 4.63%. Galantamine used as a positive control exhibited the acetylcholinesterase inhibition rates from 38.56 ± 1.83% to 77.00 ± 1.33%, when its concentrations changed from 0.008 to 0.064 μg/ml. The results showed that the IC_50_ values of PTFELDC and galantamine were 0.58 and 1.56 × 10^–5^ mg/ml, respectively. Compared with galantamine, PTFELDC displayed a weak inhibitory activity on acetylcholinesterase, which was about 3.72 × 10^4^ times lower.

## 4 CONCLUSIONS

The efficient UMSE technique was used to extract TFELDC, and a Box–Behnken design was employed to optimize the extraction process. The optimal parameters were as follows: ethanol volume fraction 71.5%, *L*/*S* ratio 12.2 ml/g, microwave power 318 W, and Extraction time 143 s. Under the optimized conditions, the experimental extraction yield achieved to 1.050%, which was well‐matched with the predicted value (1.044%), indicating that the model was adequate for the extraction process. After TFELDC were purified by AB‐8 macroporous resin, the total flavonoid contents of PTFELDC increased from 208.18 ± 1.60 to 511.19 ± 3.21 mg RE/g FDS, which was increased by 2.46 times. Thus, it can be concluded that the purification of TFELDC by AB‐8 macroporous resin is an effective method. The evaluation of the antioxidant and enzyme inhibitory activities of PTFELDC suggested that they could be developed as naturally potential antioxidants, antilipidemic, or hypoglycemic drugs for functional foods and pharmaceuticals, while the mechanism of antioxidant, antilipidemic, or hypoglycemic activities of PTFELDC in vivo need to be further elucidated. In this study, the result did not show that they were suitable to be used as an acylchoinesterase inhibitory agent, which need further discussion.

## CONFLICT OF INTEREST

There is no conflict of interest recorded for this work.

## AUTHOR CONTRIBUTION

Chao Li designed the experiments, wrote and edited the original draft, and analyzed the data; Yingbin Shen: designed the experiments, wrote and edited the original draft, and involved in formal analysis; Shanglong Chen extracted the data; Jin Sha contributed to antioxidant assay; Jue Cui analyzed the data; Juping He contributed to enzyme inhibitory activities; Junning Fu wrote—review and editing.
